# Possible role of eclosion rhythm in mediating the effects of light-dark environments on pre-adult development in *Drosophila melanogaster*

**DOI:** 10.1186/1471-213X-5-5

**Published:** 2005-02-22

**Authors:** Dhanashree A Paranjpe, D Anitha, MK Chandrashekaran, Amitabh Joshi, Vijay Kumar Sharma

**Affiliations:** 1 Chronobiology Laboratory, Evolutionary and Organismal Biology Unit, Jawaharlal Nehru Centre for Advanced Scientific Research, PO. Box. 6436, Jakkur, Bangalore-560064, Karnataka, India; 2 Evolutionary Biology Laboratory, Evolutionary and Organismal Biology Unit, Jawaharlal Nehru Centre for Advanced Scientific Research, PO. Box. 6436, Jakkur, Bangalore-560064, Karnataka, India

## Abstract

**Background:**

In insects, circadian clocks have been implicated in affecting life history traits such as pre-adult development time and adult lifespan. Studies on the *period (per) *mutants of *Drosophila melanogaster*, and laboratory-selected lines of *Bactrocera cucurbitae *suggested a close link between circadian clocks and development time. There is a possibility of clock genes having pleiotropic effects on clock period and pre-adult development time. In order to avoid such pleiotropic effects we have used wild type flies of same genotype under environments of different periodicities, which phenotypically either speeded up or slowed down the eclosion clock of *D. melanogaster*.

**Results:**

We assayed pre-adult development time and pre-adult survivorship of four laboratory populations of *D. melanogaster*, under five different light regimes, continuous light (LL), continuous darkness (DD), and light-dark (LD) cycles of 10:10 h (*T20*), 12:12 h (*T24*), and 14:14 h (*T28*). Although the development time was significantly different in most light regimes, except for females under *T24 *&*T28*, pre-adult survivorship remained largely unaffected. The development time was shortest under LL, followed by *T20*, DD, *T24 *and *T28 *regimes, in that order. Interestingly the development time showed a positive correlation with the period of eclosion rhythm, i.e., faster oscillations were associated with faster development, and slower oscillations with slower development.

**Conclusion:**

Based on these results we conclude that periodicity of imposed LD cycles, and/or of eclosion rhythm plays a key role in regulating the duration of pre-adult development in *D. melanogaster *in a manner that does not involve direct pleiotropic effects of clock genes on both clock period and development time.

## Background

Circadian (Latin: *circa *= about, *dies *= day) clocks regulate a number of physiological and metabolic processes in organisms as diverse as unicellular bacteria, fungi, fruit flies and humans [1,2]. The core molecular mechanisms underlying these rhythms are conserved across a range of taxa, and involve the expression of several clock genes, interlocked in transcriptional – translational auto-regulatory feedback loops [2].

Circadian clocks have been implicated in affecting life history traits such as pre-adult development time, and adult lifespan [3-5]. It is generally believed that faster clocks speed up pre-adult development, and shorten adult lifespan, while slower clocks slow down development and lengthen adult lifespan. The role of circadian clocks in the development of *Drosophila melanogaster *has been quite elegantly addressed in an exhaustive study on the *period *(*per*) mutants, which display circadian rhythms with widely different periods [3]. The pre-adult development time of the different *per *mutants under continuous dim light (LL) and continuous darkness (DD) was positively correlated with the free-running period (τ) of their circadian clocks, i.e. *per*^S ^mutants (τ = 19 h) developed faster than wild type flies (τ = 24 h), which in turn developed faster than *per*^*L *^mutants (τ = 28 h) [3]. The correlation between development time and clock period remained unchanged even under very bright continuous light (VLL), wherein flies are rendered arrhythmic [6,7]. Moreover, the development time and clock period showed positive correlation even under light-dark (LD) cycles of 12:12 h, and LD 12:12 h superimposed with temperature cycles (LD 12:12 T), wherein flies of different genotypes were entrained to a common 24 h periodicity. A positive correlation between development time and clock period under LD cycles is difficult to explain, unless one considers pleiotropic effects of clock genes that are not mediated by a direct causal relationship between clock period and development time. In a separate study on the melon fly, *Bactrocera cucurbitae*, which involved selection for faster and slower pre-adult development, the selection regimes yielded faster developing lines with faster circadian clocks (τ ~ 22.6 h), and slower developing lines with slower circadian clocks (τ ~ 30.9 h)[8,9]. A positive correlation between development time and clock period in the above studies suggests pleiotropic effects of clock genes on period of circadian rhythms and pre-adult development time. Therefore, it appears that the genotype, which enables the fly clocks to run faster or slower also aids faster and slower pre-adult development thus leaving the primary question of the role of circadian clocks in regulating pre-adult development in *D. melanogaster *unresolved. In order to investigate the role of circadian clocks in development time without confounding pleiotropic effects of clock genes one would need to assay development time of flies from similar genetic background under short and long day lengths, wherein their clocks would entrain by speeding up or slowing down oscillations.

In the present study we assayed pre-adult development time and pre-adult survivorship of four large out-bred laboratory populations of *D. melanogaster *(LL1.. 4) under five different light regimes: LL, DD, LD cycles of 10:10 h (*T20*), 12:12 h (*T24*), and 14:14 h (*T28*). The average periodicity of eclosion rhythm in *T20*, DD, *T24*, and *T28 *was, 20 h, 23.5 h, 24 h and 28 h, respectively, while eclosion was arrhythmic under LL [10]. The results suggest that periodicity of LD cycles and/or of eclosion rhythm play an important role in determining the duration of pre-adult development in *D. melanogaster*.

## Results

**Figure 1:**
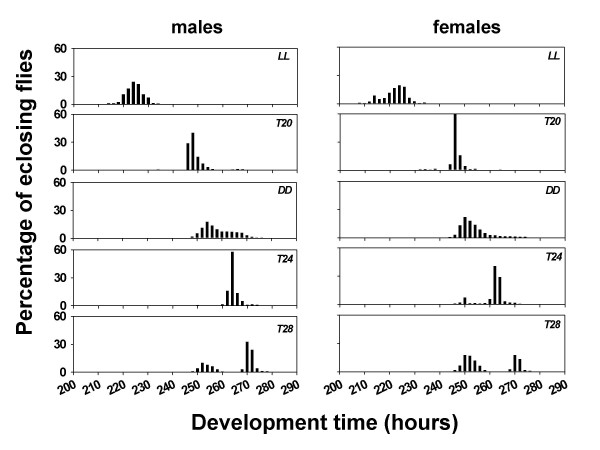
Eclosion profile of fruit flies *D. melanogaster *in five different light regimes. Pre-adult development time in hours is plotted along x-axis, and the percentage of eclosing flies is plotted along y-axis. Eclosion profile of males in five different light regimes is shown in the left panels, while that of females is illustrated in the right panels. Graphs were plotted using data pooled over four populations.

**Figure 2:**
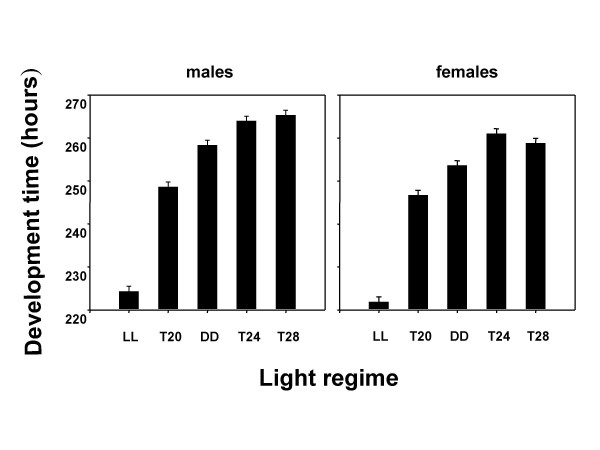
Mean pre-adult development time of *D. melanogaster *populations in five different light regimes. Light regime is plotted along x-axis, and pre-adult developmental time in hours along y-axis. Mean development time of males is shown in the left panels, while those for females in the right panels. The error bars represent 95% CI around the mean. ANOVA revealed significant effect of light regime (*F*_*4,12 *_= 2411.97, *p *< 0.001), sex (*F*_*1,3 *_= 607.85, *p *< 0.001), and light regime × sex interaction (*F*_*4,12 *_= 6.56, *p *< 0.05). The mean pre-adult development time values are provided in table1.

ANOVA on pre-adult development time data revealed a significant main effect of light regime (*F*_*4,12 *_= 2411.97, *p *< 0.001) (Figures 1, 2), while pre-adult survivorship remained largely unaffected (*F*_*4,12 *_= 1.06, *p *= 0.42). The development time of males and females was shortest under LL, followed by *T20*, DD, and *T24 *&*T28*, in that order (Figures 1, 2; see table 1). Multiple comparisons using 95% Confidence Interval (CI) around mean showed that development time of flies under different light regimes was significantly different from each other, except for *T24 *and *T28 *regimes. ANOVA also revealed a significant main effect of sex (*F*_*1,3 *_= 607.85, *p *< 0.001), and light regime × sex interaction (*F*_*4,12 *_= 6.56, *p *< 0.05). Multiple comparisons using 95% CI showed that females developed faster than males under all five light regimes and the difference in male-female development time was greatest under *T28 *regime, followed by DD, *T24*, LL and *T20 *regimes. In addition, eclosion appeared to be bimodal under *T28*, and the pattern of bimodality was more prominent in females than in males (Figure 1). In order to compare the waveform of eclosion under five light regimes, Kruskal-Wallis test was performed, and the test revealed a significant main effect of light regime on the eclosion profile of males [*H *(4, *N *= 2566) = 1923.295, *p *< 0.001] and females [*H *(4, *N *= 2709) = 2061.568, *p *< 0.001]. The Kruskal-Wallis test thus confirmed the results obtained in ANOVA. Although eclosion waveforms under no two light regimes were similar, the dissimilarity was even more striking under *T24 *and *T28*. The test revealed that mean development time of males was shortest under LL, followed by *T20*, DD, *T24*, and *T28*, in that order. While the mean development time of females followed a similar trend, it did not differ significantly between *T24 *and *T28*.

**Table 1:** Mean pre-adult development time of four laboratory populations of *D. melanogaster *assayed under five different light regimes.

**Light regime**	**Sex**	**Pre-adult development time (hours)**
LL	M	224.37
LL	F	221.91
		
DD	M	258.32
DD	F	253.63
		
T20	M	248.63
T20	F	246.72
		
T24	M	263.96
T24	F	261.07
		
T28	M	265.32
T28	F	258.83

**Figure 3:**
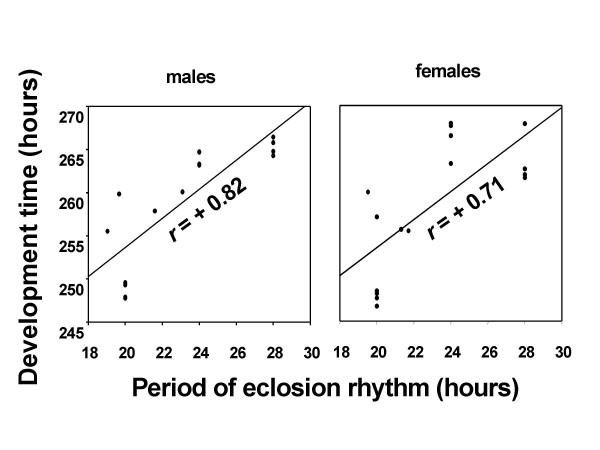
Mean development time and the period of the eclosion rhythm show a significant positive correlation. The mean development time in hours under *T20*, DD, *T24 *and *T28 *is plotted along y-axis, and the period of eclosion rhythm under the corresponding light regime is plotted along x-axis. The correlation between mean development time and period of eclosion rhythm for males is shown in left panel while that for females is illustrated in the right panel.

The periodicity of eclosion rhythm under DD, *T20*, *T24 *and *T28*, as reported in one of our previous studies on the same populations were 23.5 h, 20 h, 24 h and 28 h, respectively, while eclosion was arrhythmic under LL [10]. In addition, the peak of eclosion rhythm under different light regimes [10] matched closely the peak eclosion in the development time assay (Figure 1). The mean development time of males and females under four light regimes (DD, *T20*, *T24 *and *T28*) showed a significant positive correlation with the mean period of eclosion rhythm under the corresponding environments (*r *= +0.83 & +0.71, *p *< 0.001; Figure 3).

## Discussion

In several insect species adult eclosion is gated in a manner that it occurs only during a narrow window of time, generally around dawn when environmental humidity is the highest [11]. In *D. melanogaster *the clocks that gate adult eclosion are located in the prothoracic gland and ventral lateral neurons [12], and it is believed that these clocks also play a key role in the regulation of pre-adult development [13]. Development time under environments wherein eclosion is arrhythmic, such as bright LL, is solely determined by the developmental state of a fly, and under such a situation pre-adult development time would reflect the minimum time required by flies to complete pre-adult development. On the other hand, environments such as DD and LD cycles, wherein eclosion is rhythmic, the interaction between developmental state and eclosion clock would determine the duration of pre-adult development, and the developmental time would then be expected to be greater than those under LL. In a previous study on four *Drosophila *populations maintained under LL (JB1..4), the ancestral populations of the flies used in the present study, we had reported shortest development time under LL regime, followed by LD 12:12 h, and DD [14]. In the present study too development time was shortest under LL, followed by *T20*, DD, and *T24 *and *T28*, in that order. As opposed to LL, eclosion under DD is gated in a circadian manner, and as a result flies took longer to develop compared to that in LL. We believe that the slight discrepancy in the results of our two studies could be due to the fact that different set of flies (about 100 generations apart) with different clock periods were used in the two experiments. The periodicity of JB populations under DD was greater than 24 h, whereas those of LL populations was lesser than 24 h. Thus, consistent with our proposal, development time of JB populations was greater under DD compared to development time in LD 12:12 h regime, whereas those of LL populations was shorter in DD compared to those under LD 12:12 h.

The mean development time under four different light regimes (*T20*, DD, *T24*, and *T28*) showed a significant positive correlation with the mean period of eclosion rhythm under the corresponding light regimes; i.e. shorter period of eclosion rhythm under *T20 *was associated with faster pre-adult development, followed closely by DD, whereas flies took longest to develop under the light regimes wherein their eclosion periodicities were 24 h and 28 h, suggesting that development in *D. melanogaster *is regulated by eclosion rhythm. The peak of eclosion under three periodic light regimes closely matched phase relationships of eclosion rhythm relative to LD cycles, suggesting that phases of eclosion rhythm also play a key role in the regulation of development time [10]. Finally, flies took different number of environmental cycles to develop under three periodic light regimes. The average number of cycles taken to develop under LD 10:10 h, 12:12 h, and 14: 14 h were approximately 12.5, 11 and 9.5, respectively, which suggests that pre-adult development of *D. melanogaster *is not entirely regulated by the periodicity of the environment and/or the periodicity of eclosion rhythm. These results are in agreement with the findings of previous studies, where the duration of pre-adult development was positively correlated with clock period [3,9,15]. A subtle but important difference between the outcome of present study and that of most of previous studies is that, the correlation between developmental time and eclosion period in our study is clearly mediated via the periodicity of LD cycles and/or of eclosion rhythm, whereas in previous studies the correlation was independent of external environment and the eclosion rhythm, instead was dependent upon the genotype of the flies [3].

Although development time in the present study was greater under *T28 *compared to *T24*, the differences did not reach levels of statistical significance in ANOVA, possibly due to complex interactions between the developmental states, phase of the LD cycles, and eclosion profiles under *T24 *and *T28 *regimes. A careful analysis of development time of flies under these two regimes revealed that eclosion under *T24 *and *T28 *was bimodal, and bimodality was more prominent under *T28 *than in *T24*. To complicate the matter further the eclosion patterns of females had a greater propensity towards bimodality compared to males. The Kruskal-Wallis test revealed that eclosion profiles of flies under *T24 *and *T28 *were indeed significantly different, and mean development time of males under *T28 *was greater than in *T24 *regime, whereas those of females did not differ between the two light regimes. According to the gating hypothesis ([13]) bimodality could arise when flies are exposed to LD cycles of non-24 periodicity, perhaps due to a mismatch between developmental time and eclosion gate. Indeed, the same phenomenon also occurs under *T20*, where a small, statistically insignificant eclosion peak appears between 260 h and 270 h in both males and females (fig 1).

## Conclusion

Pre-adult development time and circadian rhythm are both multigenic traits, and genes involved in regulating development time as well as circadian rhythms are known to have pleiotropic effects [3]. Our study pre-designed to bypass such pleiotropic effects demonstrates a possible role of the periodicity of light-dark environment and/or of eclosion rhythm in determining the duration of pre-adult development. Taking into account the results of our experiments and those of the *per *mutant experiments, it appears that the duration of pre-adult development in *D. melanogaster *is determined by several factors such as the circadian rhythm, developmental state, and the interaction between the phase of eclosion rhythm, and the phase of the LD cycles.

## Methods

### Fly stock maintenance

The four populations of *D. melanogaster *used in this study were maintained under constant light (~ 100 lux), at constant temperature of 25°C (± 1°C), and constant humidity of 70 ± 5%, on a 21 day discrete generation cycle (henceforth will be referred as LL1, 2, 3, 4 populations). These populations were maintained at moderate larval densities of ~ 60–80 larvae per 8 dram vial (9.0 mm height × 2.4 mm diameter) containing banana-jaggery food medium (henceforth banana food). The ancestry and maintenance of these populations has been described in detail in an earlier paper [16]. In brief, at every generation, adults of each population are allowed to lay eggs for about 18 hours on petri plates of fresh banana food placed in a plexiglass cage (25 × 20 × 15 cm^3^). From these petri plates, 60–80 eggs are collected into each of 40 vials in which larvae then develop into adults. Adults eclosing from these vials are transferred to plexiglass cages on 12^th ^day after egg lay. On the 18th day after egg lay, adult flies are supplied with banana food supplemented with live yeast paste for two days, after which eggs are collected to initiate the next generation and the adults discarded. The breeding population typically consists of about 1500 flies.

### Pre-adult development time and survivorship assay

From the running culture of each population (LL1..4), eggs laid on banana medium over a 2 h window were collected for the assay. Exactly 30 eggs were dispensed into 8 dram vials containing ~ 6 ml banana food and 50 such vials were set up from each population. Ten vials from each population were introduced into continuous light (LL), continuous dark (DD), light-dark (LD) cycles of 10:10 h (*T20*), 12:12 h (*T24*), and 14:14 h (*T28*). Thus a total of 200 vials were set up for the assay (10 vials × 4 populations × 5 light regimes). These vials were introduced into five different light regimes at 20:00 h, when lights went off simultaneously in all LD regimes. Fluorescent white light of intensity ~ 100 lux was used during light phase, whereas in dark phase red light of λ >650 nm was used. Temperature and relative humidity in the five light environments monitored continuously using a Quartz Precision Thermo-Hygrograph, Isuzu Seisakusho Co, LTD, were found to be comparable. The vials were monitored for eclosion of adult flies after the pupae became dark. Eclosing adults were collected every 2 h, sexed, and counted until all the flies eclosed.

### Statistical analyses

Pre-adult development time in hours was calculated as the duration between the midpoint of the egg collection window and the midpoint of the 2-h period during which eclosion occurred. The mean pre-adult development time for a particular sex in a particular light regime was used as data in a mixed model analysis of variance (ANOVA), in which replicate populations were treated as random blocks, and light regime and sex were treated as fixed factors.

In order to detect differences in the eclosion profiles of the flies under different light regime, development time data of individual flies from all four replicate populations were pooled and compared using Kruskal-Wallis test.

Pre-adult survivorship was calculated as the fraction of eggs that successfully developed into adults in each vial. The mean pre-adult survivorship values of each population in each light regime were used as data in a mixed model ANOVA, with replicate populations as random blocks, and light regime as a fixed factor.

## Authors' contributions

DAP and AD were involved in collecting eggs, collecting and counting flies for pre-adult development time, estimating pre-adult survivorship, data entry and analyses. VKS conceived of the study and participated in its design and coordination. MKC and AJ gave valuable comments and suggestions throughout the study. All authors read and approved the manuscript.
